# Molecular and epidemiological characterization of human adenovirus and classic human astrovirus in children with acute diarrhea in Shanghai, 2017–2018

**DOI:** 10.1186/s12879-021-06403-1

**Published:** 2021-07-29

**Authors:** Lijuan Lu, Huaqing Zhong, Menghua Xu, Liyun Su, Lingfeng Cao, Ran Jia, Jin Xu

**Affiliations:** Department of Clinical Laboratory, Children’s Hospital of Fudan University, 399 Wanyuan Road, Shanghai, 201102 China

**Keywords:** Human adenovirus, Human astrovirus, Children, Diarrhea, Genotype

## Abstract

**Background:**

In addition to rotavirus and norovirus, human adenovirus (HAdV) and classic human astrovirus (classic HAstV) are important pathogens of acute diarrhea in infants and young children. Here, we present the molecular epidemiology of HAdV and classic HAstV in children with acute diarrhea in Shanghai.

**Methods:**

Fecal specimens were collected from 804 outpatient infants and young children diagnosed with acute diarrhea in Shanghai from January 2017 to December 2018. All of the samples were screened for the presence of HAdV and classic HAstV. HAdV and classic HAstV were detected using traditional PCR and reverse-transcription PCR, respectively. All of the HAdV and classic HAstV positive samples were genotyped by phylogenetic analysis.

**Results:**

Among the 804 fecal samples, 8.58% (69/804) of samples were infected with either HAdV or classic HAstV, and five were co-infected with two diarrhea viruses. The overall detection rates of HAdV and classic HAstV were 3.47% (28/804) and 5.22% (42/804), respectively. Four subgroups (A, B, C, and F) and seven genotypes (HAdV-C1, −C2, −B3, −C5, −A31, −F40, and -F41) of HAdV were detected. Subgroup F had the highest constituent ratio at 64.29% (18/28), followed by non-enteric HAdV of subgroup C (21.43%, 6/28) and subgroup B 10.71% (3/28). HAdV-F41 (60.71%, 17/28) was the dominant genotype, followed by HAdV-C2 (14.29%, 4/28) and HAdV-B3 (10.71%, 3/28). Two genotypes of classic HAstV (HAstV-1 and HAstV-5) were identified in 42 samples during the study period; HAstV-1 (95.24%, 40/42) was the predominant genotype, and the other two strains were genotyped as HAstV-5. No significant differences were found between boys and girls in the detection rates of HAdV (*P* = 0.604) and classic HAstV (*P* = 0.275). Over half of the HAdV infections (82.14%, 23/28) and classic HAstV infections (66.67%, 28/42) occurred in children less than 36 months. Seasonal preferences of HAdV and classic HAstV infections were summer and winter, respectively. In this study, the common clinical symptoms of children with acute diarrhea were diarrhea, vomiting, fever and abdominal pain.

**Conclusions:**

Our findings indicate that HAdV and classic HAstV play important roles in the pathogenesis of acute diarrhea in children in Shanghai. Systematic and long-term surveillance of HAdV and classic HAstV are needed to monitor their prevalence in children and prevent major outbreak.

## Background

Acute diarrhea is one of the major health problems in children under 5 years old. Approximately 1.0 billion children < 5 years of age are infected with diarrheal diseases, and 0.45 million deaths occur due to diarrhea each year [1–3]. Diarrhea can be caused by various types of viruses, bacteria, and parasites. Viruses have long been considered the most important pathogens responsible for acute gastroenteritis, with rotavirus group A and norovirus being the most prominent causes of acute diarrhea in children [4–6]. Human adenovirus and classic human astrovirus are also recognized as important causes of sporadic diarrhea and outbreaks of diarrhea in children [7, 8].

Human adenovirus (HAdV) belongs to the genus *Mastadenovirus* of the family Adenoviridae. HAdV is non-enveloped, double-stranded, 26–45 kbp linear DNA viruses that possess an outer capsid and inner core structural proteins. The outer capsid comprises fiber proteins, penton, and hexon. The fiber proteins are attached to the penton base, and penton is the second-most abundant component consisting of 12 penton bases. The hexon is the principal component of the capsid. HAdV are categorized into seven species (HAdV-A through HAdV-G) based on genomic sequence analysis, and more than 100 genotypes have been recognized [9–11]. Different genotypes have been identified by multiplex PCR techniques and sequencing of targeting fiber genes or hexon genes [12, 13]. HAdV infections lead to disease of several human systems, including acute respiratory illness, acute gastroenteritis, conjunctiva, hemorrhagic cystitis, hepatitis, hemorrhagic colitis, pancreatitis, nephritis, and meningoencephalitis [13]. Genotypes 40 and 41 of HAdV-F are the most frequently reported causes of HAdV-associated diarrhea in young children and are known as enteric HAdV. Indeed, HAdV-40 and 41 are responsible for 1–20% of diarrhea cases in both outpatients and hospitalized children worldwide [14–18]. Some cases of acute diarrhea in children have been reported to be associated with HAdV-12, − 18, and − 31 of HAdV-A. Moreover, HAdV-B, HAdV-C, HAdV-D, and HAdV-G have also been detected in fecal samples from children with acute gastroenteritis [14, 15, 18, 19].

Human astrovirus (HAstV) belongs to the *Astroviridae* family, which is divided into two genera, *Mamastrovirus* and *Avastrovirus*, based on their ability to infect mammalian and avian species, respectively. HAstV is non-enveloped, positive sense, single-stranded RNA viruses. The HAstV genome is 6.8–7.9 kb in length and consists of a 5′ untranslated region (UTR), followed by three open reading frames (ORFs) (ORF1a, ORF1b, and ORF2), a 3′ UTR, and a poly A tail. ORF1a and ORF1b encode nonstructural proteins, including the RNA-dependent RNA polymerase (RdRp), while ORF2 encodes the capsid protein precursor [20]. The initial prototype strain of the human astrovirus species was originally isolated in 1975, and is known as the classic human astrovirus (classic HAstV). With the development of next-generation sequencing technologies, two novel groups of highly divergent HAstV, Melbourne (MLB) and Virginia/Human-Mink-Ovine-like (VA/HMO), have been identified in human stools of individuals with diarrhea worldwide [7, 21, 22]. The overall detection rate of novel HAstV in stools was much lower than that of classic HAstV, which remains the second or third most common viral pathogen responsible for diarrhea in young children. Until now, eight genotypes of classic HAstV (HAstV-1 to HAstV-8) have been identified [23]. Globally, classic HAstV is responsible for 2–18.8% of cases of acute diarrhea in children. HAstV-1 is the most prevalent genotype detected in children, whereas HAstV-2–HAstV-8 are less prevalent [14, 20, 24–26].

In Shanghai, the majority of previous studies have focused on the molecular and epidemiological characteristics of rotavirus and norovirus, while relatively few studies have been conducted on the molecular epidemiology of HAdV and classic HAstV in outpatient [14, 27–29]. Therefore, we sought to investigate the detection rate, viral co-infection, seasonal distribution, age distribution, and genetic diversity of HAdV and classic HAstV infections in children with acute diarrhea in Shanghai from 2017 to 2018.

## Materials and methods

### Study design

From 2017 to 2018, a total of 804 stool specimens were collected from children < 5 years who were diagnosed with acute diarrhea and admitted to the outpatient department of the Children’s Hospital of Fudan University, Shanghai, China. All of the enrolled specimens were routinely collected and stored at − 70 °C prior to investigation. The definition of acute diarrhea was three or more loose, watery, thin stools with a paste-like texture, or the presence of mucous stools within 24 h, possibly accompanied by vomiting, abdominal pain, fever, and nausea. This definition excluded the presence of pus or blood regardless of the presence of fever [14]. Demographic information and clinical diagnoses were gathered from the children’s medical histories. Informed consent was not required from the patients because the stool specimens were collected during the normal course of patient care. The study proposal was approved by the Institutional Review Board of the Children’s Hospital of Fudan University. All methods were carried out in accordance with the relevant guidelines and regulations.

Viral genomic RNA and DNA were extracted from 10% fecal suspension supernatant using the TIANamp Virus DNA/RNA Kit (Tiangen Biotech, Beijing, China) according to the manufacturer’s instructions. Extracted genetic material was reverse transcribed into cDNA with a random primer using Prime-Script™ II Reverse Transcriptase (Takara, Biotechnology [Dalian] Co., Ltd.) for detection of the Classic HAstV. A conserved region (C4) in the HAdV hexon gene was amplified using the Ad-1 (5′-TTCCCC-ATGGCICAYAACAC-3′) and Ad-2 (5′-CCCTGGTAKC-CRATRTTGTA-3′) primers [30]. The expected size of the amplicon was 482 bp. The PCR cycling program was as follows: an initial denaturation at 94 °C for 2 min, followed by 35 cycles of 30 s at 94 °C, 30 s at 55 °C, and 1 min at 72 °C, with a final extension cycle at 72 °C for 7 min. Classic HAstV in fecal specimens was detected using primers Mon269 (5′-CAACTCAGGAAACAGGGTGT-3′) and Mon270 (5′-CTGGCTTAACCCACATTCC-3′), which targeted the ORF2 region C [31]. The expected size of the PCR product was 449 bp. PCR amplification was performed under the following conditions: 94 °C for 2 min, 35 cycles of 94 °C for 30 s, 55 °C for 30 s, and 72 °C for 1 min followed by 72 °C for 7 min. All of the PCR products were electrophoresed in a 2% agarose gel with ethidium bromide and a DNA ladder of 100 bp (Takara Bio Co., Dalian, China).

All the amplicons of HAdV and classic HAstV were purified and sequenced for phylogenetic analysis by first-generation sequencing technologies (Sangon Biotech [Shanghai] Co., Ltd.). Phylogenetic trees were constructed using the maximum likelihood method (Kimura two parameters substitution model with 1000 bootstrap replications for branch support) in MEGA (v6.0) software. The nucleotide sequences of HAdV and classic HAstV detected in this study were compared to the sequences of corresponding reference virus strains available in the GenBank database.

The nucleotide sequences of HAdV strains and the accession numbers used were as follows: HAdV-1: AC_000017, AF534906; HAdV-2: J01917, AC_000007; HAdV-3: AY599836; HAdV-4: AY487949; HAdV-5: AY339865; HAdV-8: AB448768; HAdV-9: AJ854486; HAdV-11: AY163756; HAdV-12: X73487; HAdV-14: AY803294; HAdV-16: AY601636; HAdV-17: AF108105; HAdV-21: AY601633; HAdV-22: FJ404771; HAdV-26: EF153474; HAdV-28: FJ824826; HAdV-29: AB562587; HAdV-31: AM749299; HAdV-34: AY737797; HAdV-35: AY128640; HAdV-36: GQ384080; HAdV-37: AB448777; HAdV-40: L19443; HAdV-41: DQ315364; HAdV-46: AY875648; HAdV-48: EF153473; HAdV-49: DQ393829; HAdV-53: AB605240; HAdV-54: NC 012959; HAdV-A: NC_001460; HAdV-B: NC_011203; HAdV-C: NC_001405; HAdV-D: AC_010956; HAdV-E: NC_003266; and HAdV-F: NC_001454. The reference classic HAstV strains and accession numbers used were as follows: HAstV-1: L23513, Z25771; HAstV-2: L13745; HAstV-3: AFl41381, L38505; HAstV-4: AY720891, L38506; HAstV-5: DQ028633, U15136; HAStV-6: L38507, Z46658; HAstV-7: L38508, Y08632; HAstV-8: AF260508, Z66541.

### Statistical analysis

Statistically significant differences in infection rates of categorical variables were tested using Fisher’s exact test, two-sided chi-square test and corrected chi-square test in SPSS Statistics v.20.0 (IBM Corp., Armonk, NY, USA). *P*-values < 0.05 were considered as statistically significant.

## Results

### Prevalence of HAdV and classic HAstV infections

During the study period, a total of 804 stool samples from children with acute diarrhea were enrolled in our study. Among them, 497 were boys and 307 were girls, and all of the children had been diagnosed with acute diarrhea at the Children’s Hospital of Fudan University in Shanghai following attendance as outpatients.

Among these 804 fecal samples, 8.58% (69/804) were infected with HAdV or classic HAstV, and five patients were co-infected with two viruses (Table S1 and Table S2). The overall detection rates of HAdV and classic HAstV were 3.47% (28/804) and 5.22% (42/804), respectively. The frequency of HAdV in boys and girls was 3.22% (16/497) and 3.91% (12/307), respectively (*P* = 0.604). The prevalence of classic HAstV in boys and girls was 5.84% (29/497) and 4.23% (13/307), respectively (*P* = 0.275). The prevalence of HAdV in 2017 and 2018 was 2.84% (12/423) and 4.20% (16/381), respectively (*P* = 0.293). The annual detection rates of classic HAstV varied significantly according to the year as follows: 2.84% (12/423) in 2017, 7.87% (30/381) in 2018 (*P* = 0.001).

### Seasonal and age distribution of HAdV- and classic HAstV-infected children

**Fig. 1:**
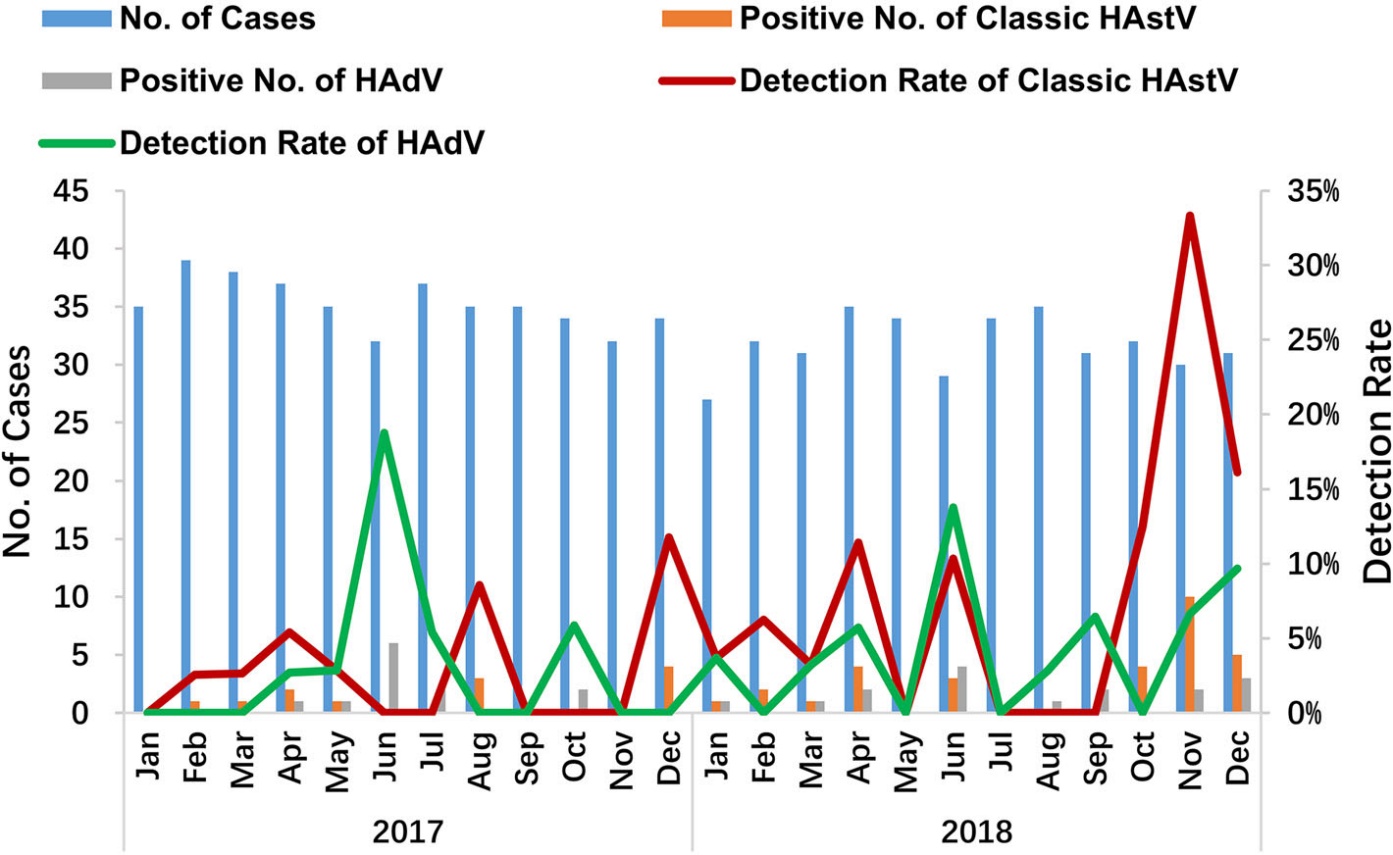
Seasonal distribution of HAdV and classic HAstV genotypes detected in the current study

The seasonal distribution of HAdV peaked in the June of both 2017 (18.75%, 6/32) and 2018 (15.38%, 4/26). During the study period, HAdV was detected in 13 of the total 24 months (Fig. 1). The peak of classic HAstV was December 2017 (11.76%, 4/34) and November 2018 (33.33%, 10/30). Classic HAstV was not detected in 10 of the total 24 months (Fig. 1).

**Fig. 2:**
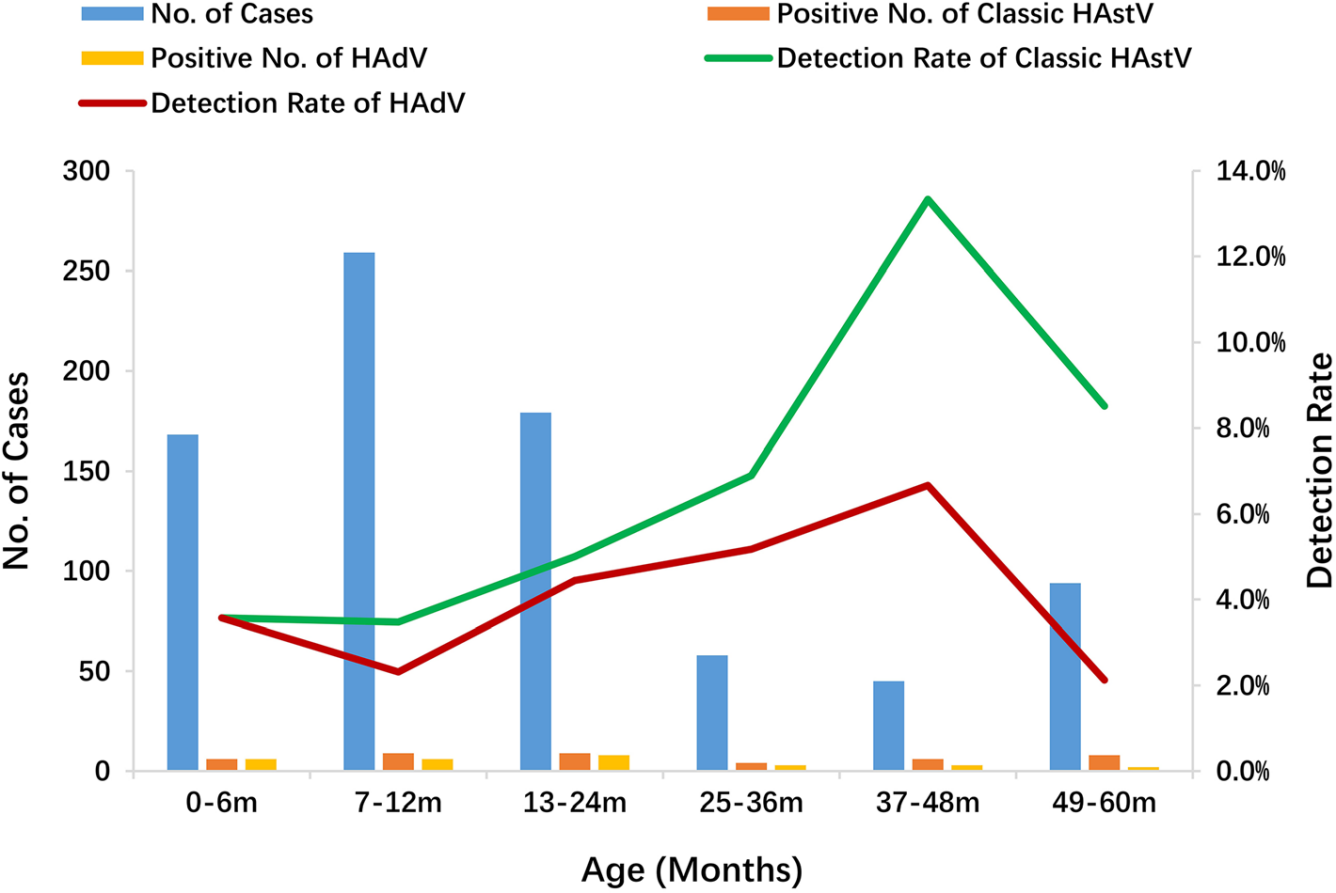
Age distribution of HAdV and classic HAstV genotypes detected in the current study

Infections of HAdV and classic HAstV were found in all age groups. Approximately 82.14% (23/28) of HAdV-infected cases and over half of classic HAstV-infected children (66.67%, 28/42) were found in children < 36 months. The group of children between 37 and 48 months old had the highest prevalence of HAdV infections (13.33%, 6/45) and HAstV infections (5.17%, 3/55) (Fig. 2).

### Genotypes of HAdV and classic HAstV infections

During the course of the study period, a total of 28 and 42 nucleotide sequences of HAdV and classic HAstV were obtained, respectively. The phylogenetic trees of nucleotide sequences of the HAdV and classic HAstV isolates were constructed in comparison to the reference strains.

**Fig. 3:**
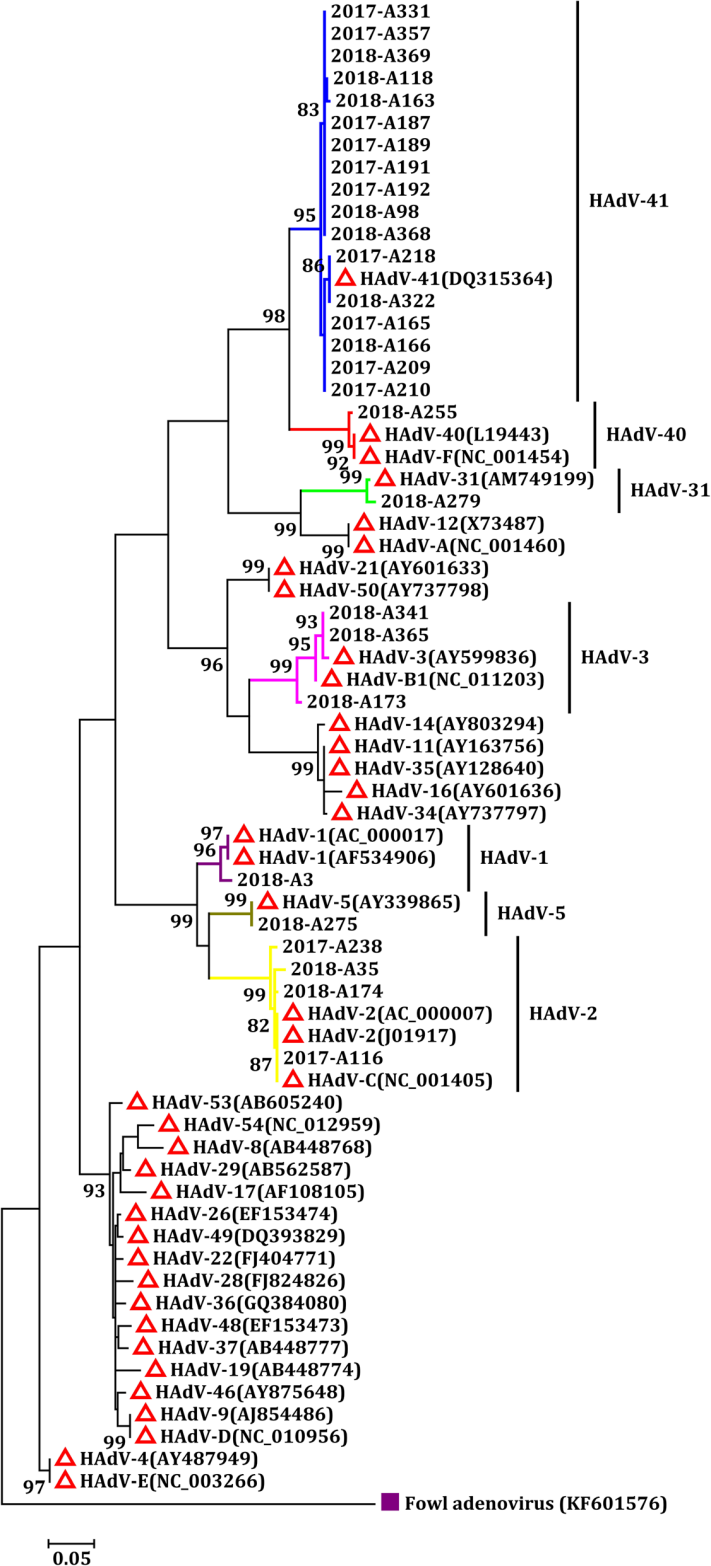
Phylogenetic analysis of partial hexon gene sequences of HAdV detected in children. Reference strains: Fowl adenovirus (KF606576)

According to the phylogenetic tree analysis conducted based on a partial genomic region of hexon, four subgroups of HAdV (A, B, C, and F) were detected, and seven different genotypes (HAdV-A31, −B3, −C1, −C2, −C5, −F40 and -F41) were identified. Subgroup F, classified as enteric HAdV, had the highest constituent ratio at 64.29% (18/28), followed by non-enteric HAdV of subgroup C (21.43%, 6/28) and subgroup B (10.71%, 3/28). Of the seven genotypes, HAdV-F41 (60.71%, 17/28) was the dominant genotype, followed by HAdV-C2 (14.29%, 4/28) and HAdV-B3 (10.71%, 3/28). HAdV-F41 was the most common genotype at 83.33% (10/12) and 43.75% (7/16) in 2017 and 2018, respectively. The second most prevalent genotype varied from 2017 to 2018. HAdV-C2 (16.67%, 2/12) was the second most prevalent genotype in 2017, while HAdV-B3 (18.75%, 3/16) was the most prevalent in 2018. Of note, only two genotypes (HAdV-C2 and HAdV-F41) were identified in 2017, while all the seven HAdV genotypes were detected in 2018 (Fig. 3).

**Fig. 4:**
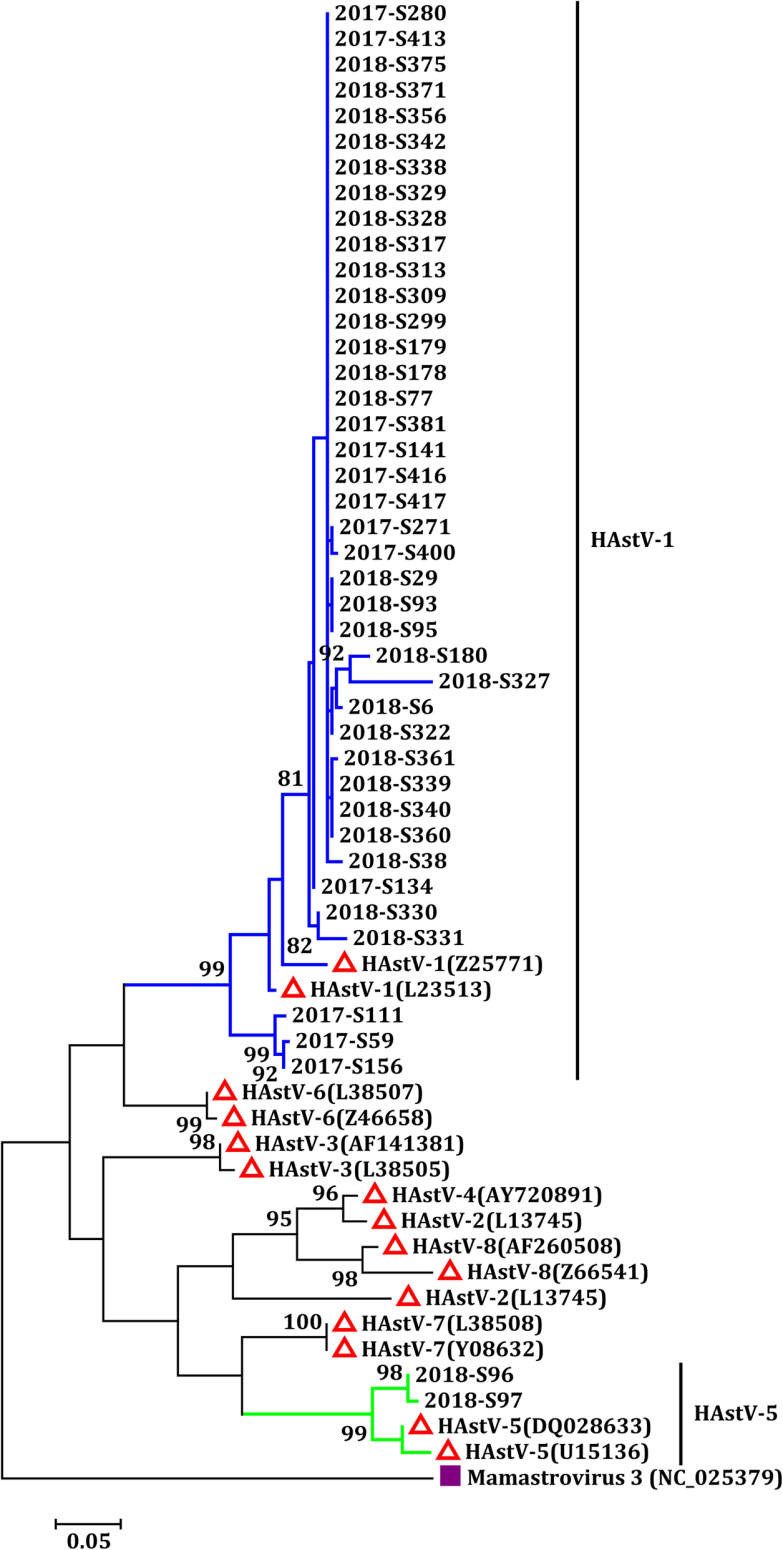
Phylogenetic analysis of partial ORF2 gene sequences of classic HAstV detected in children : Reference strains: Mamastrovirus 3 (NC_025379)

Based on the ORF2 region C of classic HAstV, two different genotypes of classic HAstV (HAstV-1 and HAstV-5) were identified in 42 samples during the study period. HAstV-1 (95.24%, 40/42) was the predominant genotype in this study and was the only genotype detected in 2017. In addition to HAstV-1 (93.33%, 28/30), HAstV-5 (6.67%, 2/30) was also identified in 2018 (Fig. 4).

### Clinical features of children infected with HAdV and classic HAstV

The most common clinical symptom of HAdV infected patients was diarrhea (100.0%, 28/28), followed by vomiting (35.7%%, 10/28), fever (25.0%, 7/28) and abdominal pain (3.6%, 1/28). The common clinical symptoms of classic HAstV infected patients were diarrhea (100.0%, 42/42), fever (26.2%, 11/42), vomiting (23.8%, 10/42) and abdominal pain (7.1%, 3/42) (Table 1). The difference between infected and uninfected classic HAstV group (*P* = 0.032) was statistically significant when the clinical symptoms of diarrhea and abdominal pain diarrhea occurred at the same time in children (Table 1). No significant differences were observed in clinical features among children with acute diarrhea under other infection circumstances (Tables 1 and 2, Tables S1 and Table S2).

**Table 1:** Clinical symptoms of diarrhea children infected with and without HAdV or classic HAstV

Clinical symptoms	HAdV, n (%)		classic HAstV, n(%)	
	Positive	Negative	Positive	Negative
Diarrhea only	13 (46.4)	486 (62.6)	20 (47.6)	474 (62.2)
Diarrhea and vomiting	7 (25.0)	114 (14.7)	8 (19.1)	120 (15.8)
Diarrhea and fever	4 (14.3)	100 (12.9)	9 (21.4)	104 (13.6)
Diarrhea and abdominal pain	1 (3.6)	16 (2.0)	**3 (7.1)**	**11 (1.4)**^a^
Diarrhea, vomiting and fever	3 (10.7)	54 (7.0)	2 (4.8)	48 (6.3)
Diarrhea, vomiting and abdominal pain	0 (0)	2 (0.3)	0	1 (0.1)
Diarrhea, fever and abdominal pain	0 (0)	1 (0.1)	0	2 (0.3)
Diarrhea, vomiting, fever and abdominal pain	0 (0)	3 (0.4)	0	2 (0.3)
Total	28 (100.0)	776 (100.0)	42 (100.0)	762 (100.0)

^a^ The difference between infected and uninfected classic HAstV group was statistically significant when the clinical symptoms of diarrhea and abdominal pain occurred at the same time (*P* = 0.032)

**Table 2:** Clinical features observed among diarrheic children infected with HAdV and classic HAstV

Clinical symptoms	Enteric HAdV, n (%)	Non-enteric HAdV, n (%)	Classic HAstV, n (%)
Diarrhea only	10 (55.5)	3 (30.0)	20 (47.6)
Diarrhea and vomiting	4 (22.2)	3 (30.0)	8 (19.1)
Diarrhea and fever	2 (11.1)	2 (20.0)	9 (21.4)
Diarrhea and abdominal pain	1 (5.6)	0 (0)	3 (7.1)
Diarrhea, vomiting and fever	1 (5.6)	2 (20.0)	2 (4.8)
Total	18 (100)	10 (100)	42 (100)

## Discussion

Although HAdV and classic HAstV generally cause a self-limiting short-term watery diarrhea, they are frequent causes of acute diarrhea in children < 5 years of age [3, 20]. Real-time monitoring of HAdV and classic HAstV can assist with monitoring their prevalence in children with acute gastroenteritis and, as a result, could play a guiding role in the prevention of major epidemics in Shanghai.

The overall stool positivity rate for HAdV infection in the present study was 3.47%, which is similar to that previously reported in Brazil (3.9%), Bangladesh (4.82%), and in our previous study (5.2%), but is much lower than that reported in Northwest Ethiopia (32.0%) and Albania (23.2%) [18, 24, 32–34]. According to our continuous monitoring data, the detection rate of HAdV in children with acute diarrheas was relatively stable in Shanghai from 2010 to 2018 [14]. In addition to the data from our previous study from 2010 to 2011 (1.9%), the detection rate of classic HAstV (5.22%) in Shanghai was also lower than the average global positive rate of 11.0% [14, 20]. This frequency is similar to that observed in other studies from Thailand (2.6%), Asian Russia (2.8%), Brazil (3.9%), Lebanon (5.5%), and Germany (5.0%) [35–39]. However, the detectable rate of classic HAstV in 2018 (7.87%) was significantly higher than the detection rate in 2017 (2.84%) in the current study, and long-term monitoring is needed to determine the reason for this increase. Furthermore, sex was not found to play a role in HAdV and classic HAstV infections in our study, which is consistent with the findings of studies in Tanzania and Northwest Ethiopia [24, 40].

Although only a small number of positive samples of HAdV and classic HAstV were reported in this study, data on the seasonality of infection with these two viruses were also analyzed. As a result, we found that HAdV and classic HAstV infections had a tendency to occur in oscillatory fluctuations. The highest rates of HAdV infection were observed in July of both 2017 and 2018, which was similar to the rates observed in Tianjin from 2008 to 2009, and Thailand from 2011 to 2017 [8, 41]. However, in our previous study on patients with acute diarrhea from 2006 to 2011, HAdV infection was more frequent during the winter months [27]. Moreover, no seasonal pattern of HAdV infection was observed in our previous study on outpatients from 2012 to 2016 [36]. Taken together, these data indicate that the seasonal pattern of HAdV infection is consistent in Shanghai. A longer time-series analysis is needed to describe the discrepancies in HAdV prevalence drawn from the acquired data of inpatients and outpatients ≤5 years of age. The same lack of seasonal pattern was found in Thailand and India [8, 42]. Similar to several other studies conducted in Germany, Spain, Northern Italy, and our previous study, classic HAstV infection was common during the cold-weather period in Shanghai [14, 26, 39, 43].

According to our data, higher HAdV (82.14%, 23/28) and classic HAstV (66.67%, 28/42) positive component ratios were identified in children ≤3 years, which is in line with the findings of other studies [19, 27, 32]. In this study, HAdV (13.33%, 6/45) and classic HAstV (6.67%, 3/42) infection were most commonly detected in children 37–48 months old. This finding suggests that herd immunity to HAdV and classic HAstV may develop gradually in children > 4 years old in Shanghai. However, the neutralizing antibody production, duration of herd immunity, and the epidemiological pattern to HAdV and classical HAstV remain to be determined.

Molecular characterization of HAdV through phylogenetic analysis revealed genetic diversity in the samples analyzed in this study. A total of seven HAdV genotypes, including five non-enteric HAdV genotypes, were found in children with acute diarrhea from 2017 to 2018. Our survey of HAdV genotypes in children with acute diarrhea indicated that enteric HAdV, including HAdV-F40 and HAdV-F41, accounted for 64.29% (18/28), and can therefore be considered the most prevalent pathogens associated with acute diarrhea in Shanghai. However, HAdV-F40 was only found in one child; this finding coincides with those from our previous studies, and those in Bangladesh and Japan [14, 32, 44]. One reason for the predominance of HAdV-F41 over HAdV-F40 is antigenic drift of HAdV-F41. Meanwhile, some studies have discovered that GTC1 and GTC2 subdivisions trigged by the build-up of amino acid mutations in the HVRs (hexon hypervariable regions) of hexon may allow HAdV-F41 to escape from the host immune response, leading to increased HAdV-F41 infection [45–47].

The results of this study suggest that non-enteric HAdV, including HAdV-A31, −B3, −C1, −C2, and -C5, play important roles in causing acute diarrhea in children, although they primarily caused conjunctiva and upper and lower respiratory tract infections [13]. Interestingly, non-enteric HAdV-C2 and HAdV-B3 infections unexpectedly exceeded that of HAdV-F40 and became the second and third leading genotype in children with acute diarrhea, respectively. In addition, in contrast to our previous studies from 2012 to 2016, the detection rate of HAdV-C2 exceeded that of HAdV-A31 and was therefore found to be the second most prevalent genotype from 2017 to 2018 [39]. Taken together, these results suggest that the genotypes of non-enteric HAdV in children with acute diarrhea undergo dynamic changes in Shanghai, demonstrating the importance of continuous surveillance of HAdV in this patient group.

HAstV-1 is the most prevalent classic HAstV genotype detected worldwide, whereas HAstV-2–HAstV-8 are less prevalent [7, 23]. According to the phylogenetic tree analysis of classic HAstV, only two genotypes, including HAstV-1 and HAstV-5, were identified in Shanghai from 2017 to 2018. HAstV-1 (95.24%) was the predominant genotype detected in children with diarrhea, which is consistent with the findings of our previous study from 2008 to 2011 as well as with other reports conducted in Japan, Switzerland, Asian Russia, Korea, Germany, and Brazil [36, 37, 39, 48–51]. Moreover, HAstV-5 was only detected in two samples in early 2018; to the best of our knowledge, this study is the first to report the appearance of HAstV-5 in Shanghai. Nevertheless, long-term monitoring data on HAstV-5 are needed to derive the epidemic characteristics of this genotype.

Typical clinical symptoms of these children with acute diarrhea were diarrhea, vomiting, fever, and abdominal pain. This finding was in agreement with the previous reports of viruses infected patients [32, 50]. Moreover, the results of this study suggest that children infected with classic HAstV would be more likely to experience abdominal pain compared with HAstV negative children.

## Conclusions

In the current study, we clarified the epidemiological role of HAdV and classic HAstV in children < 5 years with acute diarrhea in Shanghai from 2017 to 2018. HAdV-41 has a significant involvement in the etiology of acute diarrhea in children < 5 years; however, the role of non-enteric HAdV in children cannot be ignored. We also found that HAstV-1 was the most predominant genotype in Shanghai. These findings enhance our knowledge of the significance of HAdV and classic HAstV infections in children.

## Data Availability

The datasets used in the current study are available from the following link: http://purl.org/phylo/treebase/phylows/study/TB2:S27343?x-access-code=3821b7362045cab03c705697db437f13&format=html and the accession number is 27343.

## Abbreviations

HAdV: Human adenovirus
HAstV: Human astrovirus
ORFs: Open reading frames
UTR: Untranslated region
RdRp: RNA-dependent RNA polymerase
MLB: Melbourne
VA/HMO: Virginia/Human-Mink-Ovine-like
MEGA: Molecular evolutionary genetics analysis
GTC: Genome-type clusters
HVRs: Hexon hypervariable regions
